# Mitochondrial DNA in the regulation of innate immune responses

**DOI:** 10.1007/s13238-015-0222-9

**Published:** 2015-10-23

**Authors:** Chunju Fang, Xiawei Wei, Yuquan Wei

**Affiliations:** Lab of Aging Research and Nanotoxicology, State Key Laboratory of Biotherapy and Cancer Center, West China Hospital, Sichuan University and National Collaborative Innovation Center, Chengdu, 610041 China

**Keywords:** mitochondrial DNA, innate immunity, TLR9, NLRP3, STING pathway

## Abstract

Mitochondrion is known as the energy factory of the cell, which is also a unique mammalian organelle and considered to be evolved from aerobic prokaryotes more than a billion years ago. Mitochondrial DNA, similar to that of its bacterial ancestor’s, consists of a circular loop and contains significant number of unmethylated DNA as CpG islands. The innate immune system plays an important role in the mammalian immune response. Recent research has demonstrated that mitochondrial DNA (mtDNA) activates several innate immune pathways involving TLR9, NLRP3 and STING signaling, which contributes to the signaling platforms and results in effector responses. In addition to facilitating antibacterial immunity and regulating antiviral signaling, mounting evidence suggests that mtDNA contributes to inflammatory diseases following cellular damage and stress. Therefore, in addition to its well-appreciated roles in cellular metabolism and energy production, mtDNA appears to function as a key member in the innate immune system. Here, we highlight the emerging roles of mtDNA in innate immunity.

## Introduction

Microorganisms that cause disease in humans and animals enter the body at different sites and produce disease symptoms via different mechanisms. Innate immune response, or innate immunity, could immediately act as the first line of defense against various pathogens and do not involve the activation of antigen-specific lymphocytes. Innate immunity is sufficient to prevent the body from being routinely overwhelmed by a wide range of microorganisms that live on or in it (Kenneth, 2011).

Mitochondria are known as the energy factories of the cell, which are also unique mammalian organelles and considered to be evolved from aerobic prokaryotes more than a billion years ago (Dyall et al., 2004). Mitochondrion contains its own genetic material, mitochondrial DNA (mtDNA). Human mtDNA exists as a double-stranded circular loop of 16,569 bp, encoding rRNAs and tRNAs as well as 13 respiratory chain subunits (Larsson, 2010). Mitochondrial DNA, similar to that of its bacterial ancestor’s, consists of a circular loop and contains significant number of unmethylated DNA as CpG islands (Yu and Bennett, 2014). Recent research has demonstrated that mtDNA participates in different kinds of innate immune modulation, by activating molecular pathways or causing pathologies. Mounting evidence suggests that mtDNA does not only facilitate antibacterial immunity and regulate antiviral signaling, but also contribute to inflammatory diseases following cellular damage and stress. Here, we review and discuss the involvement of mtDNA in innate immune signaling pathways and the mechanisms how it contributes to the pathologies of inflammation and related disease.

## Mitochondrial DNA and pathway activation

Mitochondrial DNA contains significant number of unmethylated CpG DNA repeats that are similar to bacterial genomes. To date, several receptors and molecular pathways are reported during mtDNA function in innate immune response. The role of mtDNA as damage-associated molecular patterns (DAMPs) in inflammation initiation through Toll-like receptor 9 (TLR9) was characterized and its key role in provoking the NLRP3 inflammasome was also reported. Mitochondrial DNA also triggers stimulator of interferon genes (STING) signaling.

### A view of the mtDNA-TLR9 relationship

In recent years, the role of mtDNA as DAMPs is recognized, which are endogenous molecules released by cells undergoing abnormal cell death (e.g. during pathological insult) and capable of activating innate immune response. DAMPs could be recognized by same receptors in pathogen-associated molecular patterns (PAMPs) recognition, such as the pattern recognition receptors (PRRs). Recently, the role of mtDNA as DAMPs in inflammation initiation has gained much attention.

Bacterial DNA is recognized by TLR9, a member of the highly conserved PRRs known as TLRs (Hemmi et al., 2000). In unstimulated cells, TLR9 is located in the endoplasmic reticulum (ER). Upon stimulation by CpG DNA, TLR9 translocates to the membrane of endosomes, where they recognize their ligands and initiate cellular activation (Latz et al., 2004). Recently, mounting evidence reveals that mtDNA provokes the immune response directly via the activation of TLR9 as its ligand (Wei et al., 2015; Zhang et al., 2010). The study in our laboratory (Wei et al., 2015) has shown that cell necrosis induced by cationic nanocarriers and the resulting leakage of mtDNA could trigger severe inflammation *in vivo*, which is mediated by a pathway involving TLR9 signaling. The injection of mtDNA can activate neutrophils and increase the release of matrix metalloproteinase-8 (MMP-8), a pro-inflammatory cytokine, leading to severe inflammation in mouse lungs. Moreover, the activation of neutrophils by mtDNA through TLR9 pathway was confirmed by using TLR9^−/−^ mice and TLR9 antagonist ODN2088, and the inflammation was reduced in both TLR9^−/−^and ODN2088-treated mice (Wei et al., 2015). In addition to MMP8, interaction of TLR9 with mtDNA could activate the nuclear factor kappa B (NFκB) signaling and increase the expression of other pro-inflammatory cytokines, such as tumor necrosis factor-α (TNF-α), interleukin (IL)-6, IL-1β (Julian et al., 2013; Yu and Bennett, 2014; Zhang et al., 2014).

### Mitochondrial DNA and NLRP3 activation

The Nod-like receptor (NLR) family, pyrin domain containing 3 (NLRP3) inflammasome is the most extensively investigated one among the identified inflammasomes, which is mainly due to its ability to be activated by a wide variety of ligands (Gurung et al., 2015). Diverse stimuli, including mitochondrial damage, have been shown to provoke the NLRP3 inflammasome during infection and metabolic diseases (Horng, 2014). Bacterial and viral RNA can also activate the NLRP3 inflammasome (Kanneganti et al., 2006). Recent studies have shown that mtDNA released in the cytoplasm plays a key role in provoking the NLRP3 inflammasome (Nakahira et al., 2011; Shimada et al., 2012). Nakahira et al. showed that ATP-mediated mtDNA release depends on the NLRP3 inflammasome and mitochondrial reactive oxygen species (mROS); thus mtDNA amplifies inflammasome activation after the initial trigger (proposed to be mROS production). By contrast, Shimada et al. further suggested that the oxidized mtDNA directly bound to NLRP3 and activated the inflammasome, suggesting that it initiated activation of this pathway. Depleting mitochondria of mtDNA (by ethidium bromide treatment) impaired inflammasome activation and further supported the direct role of mtDNA in inflammasome activation (Nakahira et al., 2011). Interestingly, NLRP3 is required for the translocation of mtDNA into the cytosol during inflammasome activation (Nakahira et al., 2011). This demonstrates that cytosolic mtDNA can be identified as a putative ligand of the NLRP3 inflammasome and mtDNA might act in a positive feedback loop to potentiate the NLRP3 inflammasome activation (Gurung et al., 2015; Horng, 2014).

The NLRP3 inflammasome is a cytosolic complex, in which interaction with the adaptor protein Asc and procaspase-1 enables the recruitment and activation of caspase-1, leading to the maturation of IL-1β and IL-18 and the induction of pro-inflammatory cell death of sentinel cells in the innate immune system (Horng, 2014; Mariathasan et al., 2006; Martinon et al., 2006). Mitochondrial DNA also plays a key role in the activation of the NLRP3 inflammasome and mediates the secretion of IL-1β and IL-18 (Yu and Bennett, 2014).

### Mitochondrial DNA and the STING pathway

**Figure 1:**
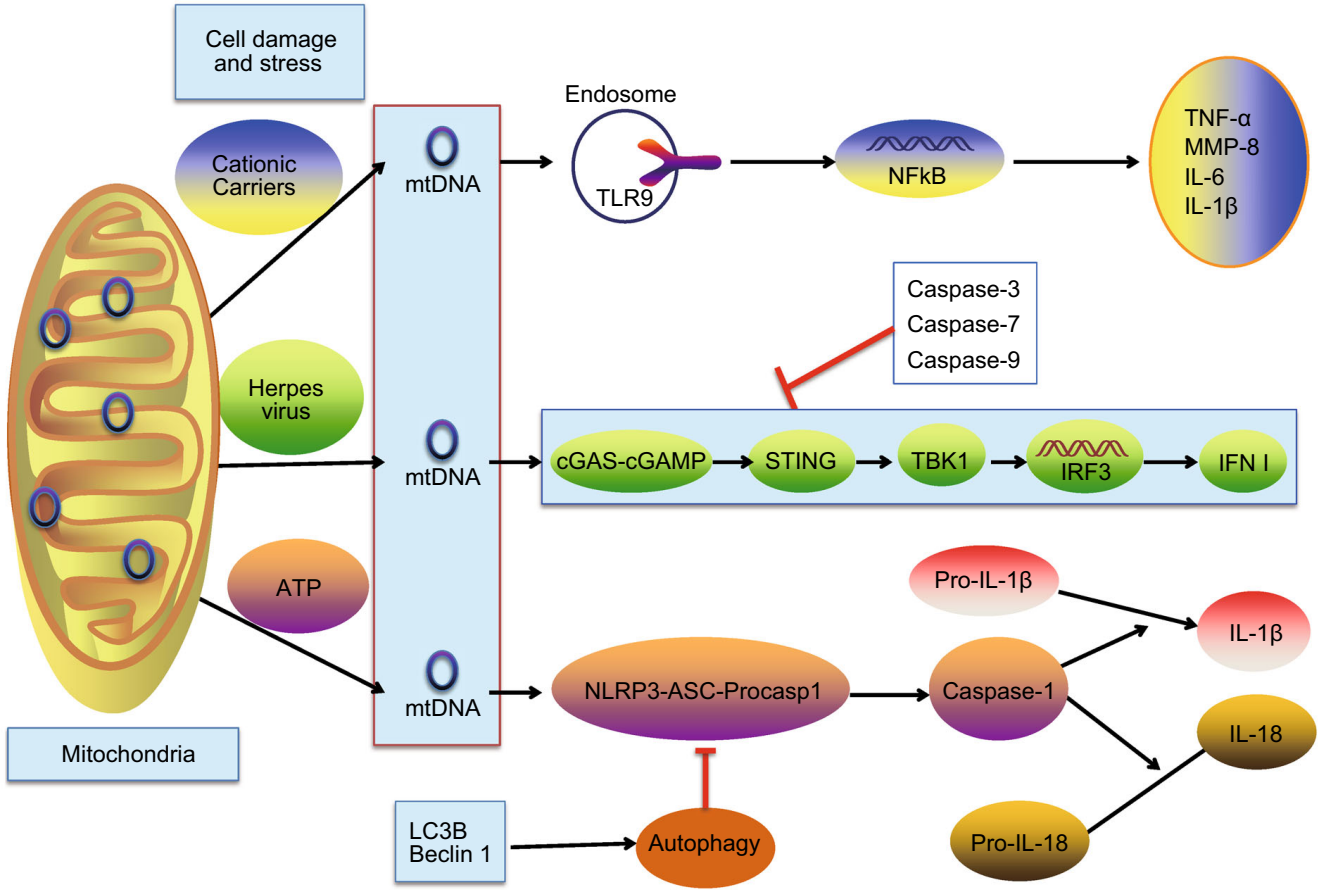
**Mechanisms by which mitochondrial DNA activates innate immunity**. Mitochondrial DNA (mtDNA) released from mitochondria following cell damage and stress (e.g. cationic carriers) can activate TLR9 in endosomes, leading to the transcription of pro-inflammatory cytokine genes and increased release of pro-inflammatory cytokines, including MMP-8, TNF-α, IL-6 and IL-1β. In addition, mtDNA escaped into the cytosol after herpes virus infection, can be detected by the cGAS-cGAMP-STING pathway, which results in TBK1-IRF3-dependent expression of type I interferon (IFN I) and dampening viral replication. However, the activation of caspases involved in the intrinsic pathway of apoptosis (caspase-3, caspase-7 and caspase-9) can prevent the activation of IFN response. Moreover, stimulation such as ATP induces mitochondrial dysfunction resulting in mtDNA release into the cytoplasm, where it binds to and activates the NLRP3 inflammasome. Interaction with the adaptor protein Asc and procaspase-1, the NLRP3 inflammasome enables the recruitment and activation of caspase-1, which cleaves pro-IL-1β and pro-IL-18 into their bioactive mature forms. On the other hand, microtubule-associated protein 1 light chain 3B (LC3B)/Beclin 1-mediated autophagy are involved in the clearance of mtDNA, and thus negatively regulating the NLRP3 inflammasome activation

In addition to interacting with the TLR9 pathway and the NLRP3 inflammasome, mtDNA can also activate the STING pathway. A recent study (West et al., 2015) has showed that mitochondrial transcription factor A (TFAM) depletion, induced genetically or during herpesvirus infection, triggers disruption of mtDNA stability, which is characterized by nucleoid loss and enlargement. This results in the release of fragmented mtDNA that recruits and activates peri-mitochondrial cyclic GMP-AMP synthase (cGAS) to generate the second messenger cyclic GMP-AMP dinucleotide (cGAMP) and activate endoplasmic-reticulum-resident STING. STING then activates TANK-binding kinase 1 (TBK1), which phosphorylates interferon regulatory factor 3 (IRF3) and results in IRF3-dependent expression of type I interferon (IFN I) and other interferon-stimulated genes and augments viral resistance (summarized in Fig. 1). Both responses are found in innate antiviral defenses to dampen viral propagation. In summary, herpesvirus infection induces mtDNA stress, which leads to the activation of antiviral innate immune responses through the cGAS-STING pathway. Another two recent studies (Rongvaux et al., 2014; White et al., 2014) demonstrate that Bak- and Bax- mediated mitochondrial damage in the absence of activating the downstream apoptotic caspases induces the release of mtDNA, thereby triggering cGAS-cGAMP-STING signaling.

To conclude, mtDNA participates in a variety of innate immune pathways, including mtDNA-TLR9-NFκB axis, mtDNA-NLRP3-caspase1 pathway and mtDNA-STING-IRF3 signaling (summarized in Fig. 1), leading to diverse innate immune responses subsequently mentioned.

## Mitochondrial DNA and innate immunity

### Mitochondrial DNA and antiviral responses

Although known as cellular powerhouses, mitochondria also regulate programmed cell death pathways and function as centrally positioned hubs in the innate immune system. Recently, mitochondria were shown to help elicit cellular inflammation, particularly by inducing antiviral signaling pathways (West et al., 2011). The cytosolic sensors Retinoic acid- inducible gene I (RIG-I) and melanoma differentiation-associated gene 5 (MDA5) recognize distinct viral RNA species and activate signaling through mitochondrial antiviral signaling (MAVS), which results in the expression of cytokines including type I and III interferon to restrict viral replication (Yoneyama et al., 2015). In addition to mitochondria, mtDNA can also contribute to antiviral immunity via the mtDNA-STING-IRF3-IFN I signaling as mentioned above. Mitochondrial DNA can be identified as a cell-intrinsic trigger of antiviral signaling and cellular monitoring of mtDNA homeostasis cooperates with canonical virus sensing mechanisms to fully provoke antiviral innate immunity (West et al., 2015).

### Mitochondrial DNA and antibacterial immunity

It has been reported that mitochondria has the ability to facilitate antibacterial immunity by generating ROS and ROS derived from mitochondria plays a key role in macrophage-associated antibacterial immune responses (West et al., 2011). Such as mROS, mtDNA also participates in antibacterial responses. Certain host immune cells (e.g. neutrophils and mast cells) use nuclear DNA-based extracellular traps (ET) (consisting of nuclear DNA, histones and antimicrobial peptides, etc.) to catch microbial pathogens and kill them (von Kockritz-Blickwede and Nizet, 2009). It has been shown that eosinophil can eject mtDNA specifically, which functions as an ET to assist in immobilization of the microbial pathogens, and thus, the eosinophil can recognize and kill the microorganism (Yousefi et al., 2008). Like neutrophil ET and mast cell ET, the eosinophil ET process is dependent on ROS production, but importantly, does not result in cell death since the eosinophils remain viable after ejection of its mtDNA. Recently, Morshed and colleagues (Morshed et al., 2014) observed that both human and mouse basophils are able to produce mROS and form extracellular mtDNA traps upon IL-3 priming in the NADPH oxidase-independent form. Another recent article (Yousefi et al., 2015) provides evidence that, in spite of an apparent lack of phagocytic activity, basophils can kill bacteria through basophils extracellular traps (BETs) formation, which contains mtDNA and granule proteins. As mentioned above, the pathogen-induced mtDNA-based ETs in eosinophils and basophils have important roles in antibacterial immunity.

In summary, a pathogen-induced mitochondrial stress, leading to the release of mtDNA, is an evolutionarily beneficial mechanism in the host that amplifies antibacterial and antiviral signaling in response to pathogen invasion. However, the aberrant accumulation of damaged mitochondria and the leakage of mtDNA into the cytosol may also cause inflammatory diseases (Kanneganti et al., 2015).

### Mitochondrial DNA and inflammatory diseases

It is reported that mtDNA can be detected in the synovial fluids of rheumatoid arthritis (RA) patients but not in healthy individuals (Collins et al., 2004), which is also increased in the plasma of patients with femur fracture (Zhang et al., 2010) or with acute human immunodeficiency virus (HIV) infection (Cossarizza et al., 2011). Mitochondrial DNA can activate polymorphonuclear neutrophils through CpG/TLR9 interactions in sterile systemic inflammatory response syndrome (SIRS) associated with acute trauma (Zhang et al., 2010). The study in our laboratory (Wei et al., 2015) has shown that mtDNA released from necrotic cells induced by cationic carriers can mediate the inflammatory responses via TLR9 signaling, which reveals a novel mechanism about the inflammatory toxicity of cationic carriers and provides a new vision of designing better and safer cationic carriers for drug delivery. Moreover, pressure-overload released mtDNA that escapes autophagy causes inflammatory responses in cardiomyocytes, and is capable of inducing myocarditis and dilated cardiomyopathy (Oka et al., 2012). Mounting evidence reveals that mtDNA is thought to play a key role during sterile inflammation in the heart (Nakayama and Otsu, 2013). Despite RA, SIRS and heart diseases, mtDNA also participates in systemic inflammation during acute liver failure (Marques et al., 2012), Parkinson’s disease (Celardo et al., 2014) and atherosclerosis (Ding et al., 2013) via TLR9-mediated inflammatory responses. To conclude, these data indicate that mtDNA has a vital role in DAMP-associated inflammation in different pathological disorders.

It has been demonstrated that the NLRP3 pathway participates in a variety of important responses such as host defense, where its activity is beneficial, and the NLRP3 pathway also contributes to metabolic diseases, where it may play a pathophysiological role (Franchi et al., 2009; Lamkanfi and Dixit, 2012; Strowig et al., 2012; Wen et al., 2013). Similarly, mtDNA also plays a key role in the activation of the NLRP3 inflammasome and mediates the secretion of IL-1β and IL-18, which might be associated with the induction of inflammatory disease. It is reported that mtDNA damage may lead to mitochondrial dysfunction and increase the secretion of IL-1β, which directly promotes atherosclerosis (Yu and Bennett, 2014). What’s more, the interactions between mROS/mtDNA and the NLRP3 inflammasome have the ability to facilitate IL-1β release and pancreatic β-cell death and contribute to type-2 diabetes mellitus progression (Escames et al., 2012; Nishikawa and Araki, 2007).

## Conclusions and perspectives

Mitochondrial DNA, activation of TLR9, NLRP3 and STING, is recently discovered and investigated as a key modulator in innate immune signaling (summarized in Fig. 1). Mitochondrial DNA, but not nuclear genomic DNA, generally released in the cytoplasm after cellular damage and stress, plays an important role in the development of different kinds of inflammatory diseases, including RA, sterile SIRS, acute liver failure, atherosclerosis, heart diseases and Parkinson’s disease, as well as in antibacterial immunity and antiviral signaling (Collins et al., 2004; Zhang et al., 2010; Marques et al., 2012; Yu and Bennett, 2014; Oka et al., 2012; Celardo et al., 2014; Yousefi et al., 2015; West et al., 2015). Two recent articles (Rongvaux et al., 2014; White et al., 2014) have highlighted the role of mtDNA sensing by the cGAS-STING pathway. They concurrently published that the activation of caspases involved in the intrinsic pathway of apoptosis (caspase-3, caspase-7 and caspase-9) can prevent the activation of IFN response mediated by the release of mtDNA in cells undergoing apoptosis caused by Bax and Bak. In addition, microtubule-associated protein 1 light chain 3B (LC3B)/Beclin 1-mediated autophagy are involved in the clearance of damaged mitochondria, and thus negatively regulate the NLRP3 inflammasome activation (Nakahira et al., 2011). Two recent studies reported that the γ-aminobutyric acid A receptor-associated protein (Gabarap) and α7 nicotinic acetylcholine receptor (α7 nAchR), respectively, are involved in mitochondrial quality control in macrophages, and their deficiency enhance the mtDNA/NLRP3 dependent inflammatory responses (Lu et al., 2014; Zhang et al., 2013). The discovery of the molecular pathways that mtDNA activates while it is recognized by innate immune cells might provide us with new targets for treatment of the related diseases.

However, several questions remain. First, are there more mtDNA pathways in innate immunity that are not discovered? Second, in addition to above-mentioned diseases, what are the other diseases, such as cancer, related with mtDNA? Last but not least, what mechanisms exist in the process of mtDNA being released from the mitochondria? One study suggests that mtDNA release is mediated by mitochondrial permeability transition (MPT) based on sensitivity to cyclosporine A, which is plausible if MPT is followed by osmotic swelling and mitochondrial rupture (Nakahira et al., 2011). A recent study demonstrates that the hydrolysis of the mitochondrial membrane by secreted phospholipase A2 IIA (sPLA2-IIA) yields inflammatory mediators (e.g. lysophospholipids and mtDNA) that promote leukocyte activation, leading to inflammatory responses (Boudreau et al., 2014). It is important to explore more mechanisms between mtDNA and the innate immune responses.
